# Lipopolysaccharide from Rhodobacter spheroids modulate toll-like receptors expression and tissue damage in an animal model of bilateral renal ischemic reperfusion injury

**DOI:** 10.25122/jml-2021-0255

**Published:** 2022-05

**Authors:** Munaf Aal-Aaboda, Ahmed Rahma Abu Raghif, Rihab Hameed Almudhafer, Najah Riesh Hadi

**Affiliations:** 1. Department of Pharmacology, Faculty of Pharmacy, University of Misan, Amarah, Iraq; 2. Department of Pharmacology, Faculty of Medicine, Al-Nahrain University, Baghdad, Iraq; 3. Middle Euphrates Unit for Cancer Research, Faculty of Medicine, University of Kufa, Kufa, Iraq; 4. Department of Pharmacology and Therapeutics, Faculty of Medicine, University of Kufa, Kufa, Iraq

**Keywords:** ULPS-RS, *Rhodobacter Sphaeroides*, LPS-RS, TLR2, TLR4, pure TLR4 antagonist, mixed TLR2, TLR4 antagonist

## Abstract

Ischemic reperfusion injury (IRI) of the kidneys is a direct sequela of surgical procedures associated with the interruption of blood supply. The pathophysiology of IRI is complicated, and several inflammatories, apoptosis, and oxidative stress pathways are implicated. Among the major receptors directly involved in renal IRI are the toll-like receptors (TLRs), specifically TLR2 and TLR4. In this study, we investigated the effects of Lipopolysaccharide from *Rhodobacter Sphaeroides* (TLR2 and TLR4 antagonist, LPS-RS) and the ultrapure form (pure TLR4 antagonist, ULPS-RS) on the histopathological changes and TLRs expression in an animal model of bilateral renal IRI. Forty-eight adult male rats were allocated into six groups (N=8) as follows: sham group (negative control without IRI), control group (rats underwent bilateral renal ischemia for 30 minutes and 2 hours of reperfusion), vehicle group (IRI+ vehicle), LPS-RS group (IRI+ 0.5 mg/kg of LPS-RS), ULPS-RS group (IRI+ 0.1 mg/kg of ULPS-RS), ULPS-RSH group (IRI+ 0.2 mg/kg of ULPS-RS). Significant improvement in the histopathological damages induced by renal IRI was found in the ULPS-RS treated groups at both doses compared with the control group. The protective effect of ULPS-RS was associated with significantly reduced TLR4 expression without affecting TLR2. Regarding LPS-RS, the tested dose adversely affected the renal tissues as manifested by the histopathological findings, although it similarly affected TLRs expression as ULPS-RS. Our results demonstrated that ULPS-RS was renoprotective while LPS-RS had no protective effect against the tissue damages induced by renal IRI.

## INTRODUCTION

Chronic kidney disease is among the major diseases affecting around 10% of the population with increasing prevalence due to the higher incidence of diabetes and hypertension, regarded as the twelfth leading cause of death in 2015 [1]. The ultimate consequence of chronic kidney disease is end-stage renal disease, with its optimal therapy being kidney transplantation because it results in a better quality of life than dialysis. A major sequela of renal transplantation is the renal ischemic-reperfusion injury (IRI), the main pathological mechanism mediating graft rejection and dysfunction following renal transplantation [2–4]. Moreover, IRI is also considered the leading cause of acute kidney injury (AKI), affecting around thirteen million worldwide, with roughly 1.7 million deaths each year [5]. Renal IRI is a critical medical condition that occurs due to transient impairment of blood flow to the kidneys with the subsequent restoration of blow flow and re-oxygenation [6]. The causes of renal IRI include partial nephrectomy, cardiac surgeries with clamping of the aorta, sepsis, shock, and trauma [7, 8]. The clinical consequences of renal IRI range from mild kidney impairment to severe clinical conditions, assuring the need for dialysis or transplantation, according to the magnitude of the injury [9].

Furthermore, IRI is among the primary causes of mortality and morbidity [10], and it constitutes an important risk factor for progression to chronic kidney disease [11, 12]. A large pool of studies was performed to elucidate the abnormal events underlying the IRI of the kidneys. Different pathologic processes have been postulated as essential in the pathogenesis of renal IRI, like dysfunction of renal tubular epithelial cells, disturbances in the renal microcirculation, inflammatory reaction, loss of endothelial integrity, and the synthesis of reactive oxygen species [6]. In brief, the ischemic phase precipitates dysfunction of the endothelial layer of the glomerular capillaries and necrotic events affecting the tubular epithelial cells, followed by reperfusion-induced massive generation of reactive oxygen species. A robust inflammatory reaction is involved in both phases[13]. Unfortunately, the subsequent reperfusion exacerbates the damage, activating numerous mechanisms involving the cell death programs and the adaptive and innate immune responses [14]. Among the most important receptors that participate in coordinating the inflammatory response in renal IRI are TLRs. Previous studies showed nephroprotective effects following antagonism of TLR4, TLR2 and both of them [15–17]. Accordingly, we aimed to investigate the effect of the pure TLR4 antagonist ultrapure lipopolysaccharide from *Rhodobacter Sphaeroides* (ULPS-RS) and the proposed mixed TLR4 and TLR2 antagonist which is lipopolysaccharide from *Rhodobacter Sphaeroides* (LPS-RS) on renal IRI and to explore their effects on TLRs expression in an animal model of bilateral IRI of the kidneys.

## MATERIAL AND METHODS

### Experimental animals

In this study, 48 adult male Wistar Albino rats with weights in the range of 250–350 g were included. The rats were accustomed to controlled conditions (25°C temperature; 60–65% humidity; 12-hour light-dark cycle). All experimental rats had ad-lib access to food and water. All experimental and animal housing procedures were ethically approved by the Institutional Animal Care and Use Committee at the University of Kufa.

### LPS-RS and ULPS-RS

Both compounds were purchased from Invivogen, USA (LPS-RS, Catalog #tlrl-rslps; ULPS-RS, Catalog #tlrl-prslps). The compounds were dissolved in endotoxin-free distilled water as recommended by the company on the day of the experiment.

### Animal model

Surgical induction of bilateral renal IRI was performed in accordance with a previous study [18]. Rats were weighed and anesthetized with a combination of 100 mg/kg ketamine and 10 mg/kg xylazine [19], and both drugs were given by intraperitoneal (i.p.) injection. Later, an abdominal incision was performed, exposing the renal pedicles. Bilateral renal ischemia was done by clamping the pedicles for 30 minutes. After 30 minutes, reperfusion was performed by removing the clamps, and then the incision site was closed with surgical sutures, and the rats were returned to their cages for 2 hours [20]. Later, animals were euthanized, and blood samples and the kidneys were collected [21].

### Study design

The rats used in this study were randomly divided into six subgroups (N=8) that were treated as follows: (1) sham group: the rats were anesthetized, and only laparotomy was done; (2) control group: bilateral renal ischemia was induced by clamping renal pedicles for 30 minutes followed by 2 hours of reperfusion; (3) vehicle treated group: rats in this group were treated with i.p. injection of pyrogen-free D.W 1 hour before the induction of bilateral renal IRI; (4) LPS-RS group: the rats were injected intraperitoneally with LPS-RS at a dose of 0.5 mg/kg of body weight one hour before inducing the bilateral IRI [22, 23]; (5) ULPS-RS group: one hour before inducing the bilateral renal IRI, the rats received an i.p. injection of ULPS-RS at a dose of 0.1 mg/kg [22–24]; (6) ULPS-RSHgroup: to see if this compound has dose dependent effect, the rats in this group were given i.p. injection of ULPS-RS at a dose of 0.2 mg/kg about one hour before inducing the bilateral renal IRI [22].

### Histopathological study

The left kidneys were cut into two halves. The first halves were fixed in 10% neutral buffered formalin and later used for the histopathological study that includes immunohistochemistry staining (IHC-P) along with hematoxylin and eosin staining (H&E). The fixed renal tissues were transferred into a fully automated Leica tissue processor to prepare tissue blocks. Later, a microtome was used to obtain tissue slices of 4 µum thickness. These slices were mounted into glass slides and stained with H&E as previously described [25]. These stained slides were then examined using a blind method by histopathologists to evaluate renal tissue injury. The damage to renal tissues was defined as swelling of tubular epithelial cells, brush border loss, cast formation, necrotic tubules, vacuolar degeneration, and desquamation. The magnitude of tubular injury was examined in five randomly selected fields and scored as follows: 0, normal; 1, less than 25% of tubules are damaged; 2, 25–50% of tubules are damaged; 3, 50–75% of tubules are damaged; and 4, more than 75% of tubules are damaged [26].

### Immunohistochemistry staining

Positively charged slides were prepared to form the paraffin-embedded tissue blocks of the left kidneys and were used for IHC-P staining for TLR2 and TLR4. The IHC-P staining was performed according to the labeled Streptavidin-biotin method using the Dako Envision Flex High pH link kit [27]. Briefly, the slides were first heat-treated in the oven at 60°C for 60 minutes, followed by xylene deparaffinization. The tissues were then rehydrated and placed in a retrieval solution in a water bath for 20 minutes at 95°C. Later, the slides were washed with a 20X wash buffer, and then peroxidase blocking solution was added for 10 minutes, then washed. The slides were then incubated overnight with 1:200 dilution of primary antibodies for TLR4 and TLR2 (Bioassay technology laboratory). Later, the slides were washed, and a mixture of horseradish peroxidase and goat secondary antibody against rat immunoglobulin was added for 30 minutes, followed by washing. Chromogen was then added for 20 minutes, followed by washing and counterstaining with hematoxylin. Finally, the slides were washed and placed in 100% ethanol. Positive control (rat spleen tissue) was stained with primary antibody, and negative control was the same tissue without staining. Both were included in each run to confirm the specificity of the antibody. The protein expression of TLR2 and TLR4 was calculated by multiplying the percent and intensity of staining, and the result is called the Q score, which can have any value from 0 to 300. The staining intensity was scored as 0, no staining; 1, weak staining; 2, moderate staining; and 3, strong staining. The percent of stained cells was recorded from 0–100% [27–29].

### Statistical analysis

An independent sample Kruskal-Wallis test was done for the renal tissue injury score and IHC staining results, followed by Dunn's multiple comparison tests to clarify the statistical differences among the six groups [30]. For simplicity in displaying the pairwise comparisons, letters (a, ab, b, bc, c etc) were given for the corresponding average ranks in descending order. Average ranks with two letters show no significant difference from either of the average ranks with individual letters (ab is not significantly different from a or b). Figures were done using SPSS version 21. A P-value less than 0.05 was considered statistically significant.

## RESULTS

### Histopathological findings

**Figure 1:**
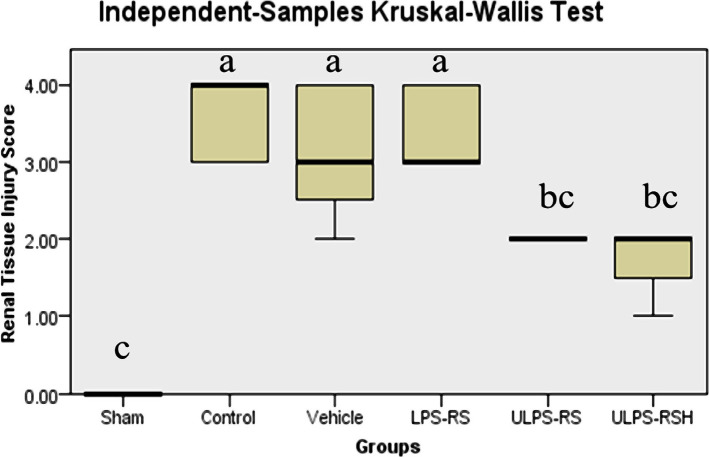
Boxplot showing the distribution of injury scores across each group. Boxes with different letters are significantly different (P<0.05).

To evaluate the differences between the experimental groups, the renal tissue injury scores were statistically analyzed. The Kruskal-Wallis test revealed significant differences in the histopathological score between the different groups (test statistics=39.913; P<0.05), and the mean ranks for the groups are shown in Table 1. The post hoc test revealed significantly higher scores in the control, vehicle, and LPS-RS groups compared with the sham group. On the other hand, the scores in the ULPS-RS groups were significantly lower than that of the control group (Table 1, Figure 1). The histological differences between the individual groups are summarized below.

**Table 1:** Summary of Dunn's post hoc multiple comparison test shows the average rank for each group.

Groups	TLR2 Q score Average Ranks	TLR4 Q Score Average Ranks	Tissue Injury score Average Ranks
**Sham**	4.69b	25.38ab	4.50c
**Control**	32.69a	39.12a	38.88a
**Vehicle**	25.31a	41.50a	32.75a
**LPS-RS**	20.06ab	17.31b	36.12a
**ULPS-RS**	34.19a	14.12b	18.50bc
**ULPS-RSH**	30.06a	9.56b	16.25bc

Means with a different letter in the same column are significantly different (P<0.05).

### Sham group

**Figure 2:**
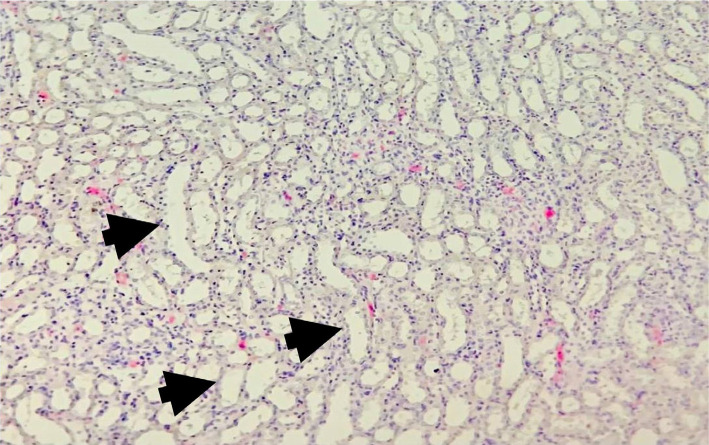
Representative photomicrograph of the sham group shows normal kidney structure (black arrows) H&E Magnification x100.

The renal tissues collected from all the sham rats demonstrated normal kidney histology with normal renal tubules and glomeruli. The brush borders were intact, and no changes were found in the interstitium and the tubular cells. Based on the used scoring system, the average score for the sham group was low (score=0, meaning none of the tubules were affected), which means normal kidneys (Figure 2).

### Control group

**Figure 3:**
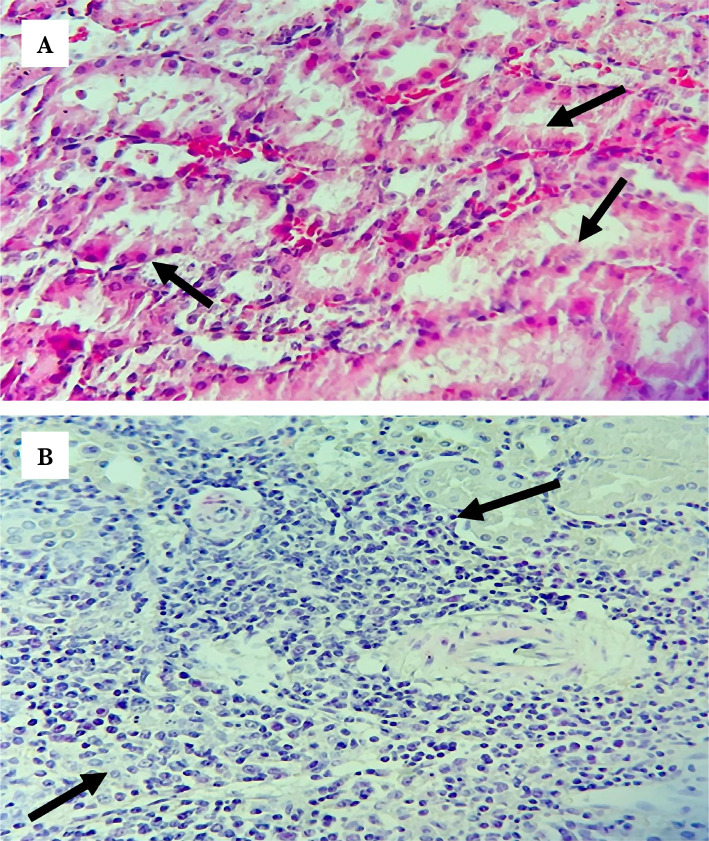
Representative photomicrographs of the control group shows (A) severe damage with prominent cellular swelling and cytoplasmic eosinophilic (black arrows), (B) heavy inflammatory cells infiltration (black arrows) H&E Magnification x400.

The histopathological findings of the control group exhibited abnormal structure and significantly elevated tissue injury scores compared with the sham group (Figure 1). Briefly, tissue damages were severe, with prominent cellular swelling and heavy infiltration by inflammatory cells found in the investigated renal tissues from all rats within the control group (Figure 3 AB). The tissue injury score was approaching 4, which means that around 75% or more of the renal tubules were affected (Figure 1).

### Vehicle group

**Figure 4:**
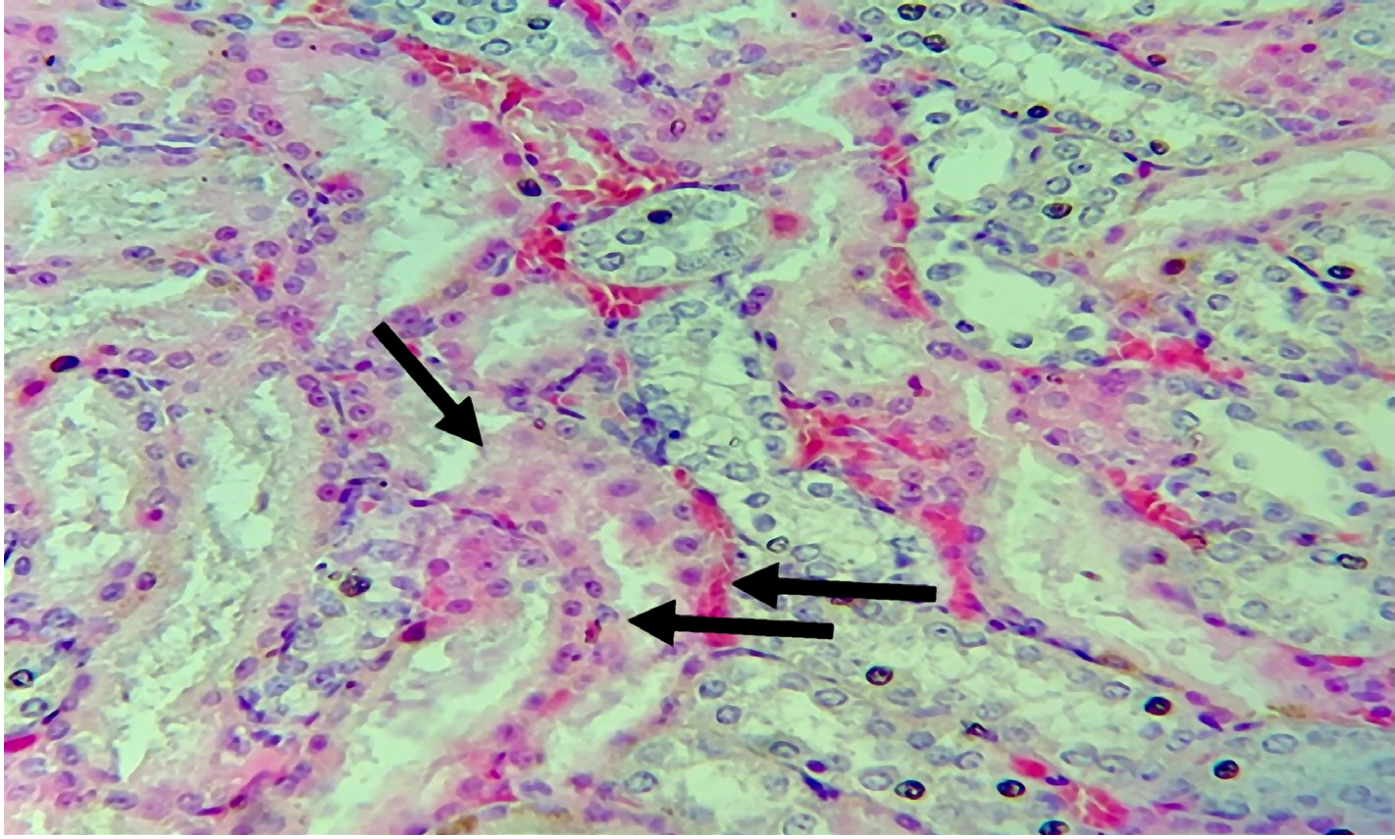
A representative photomicrograph of the vehicle group shows cellular swelling and cytoplasmic eosinophilic (black arrows), H&E Magnification x400.

Similar abnormality in the histopathological findings was documented in the vehicle group compared with the control group. Severe damages in the examined tissues were prominent cellular swelling, interstitial inflammation, and cytoplasmic eosinophilic (Figure 4). The injury scores were comparable to those of the control group (Table 1, Figure 1).

### LPS-RS group

**Figure 5:**
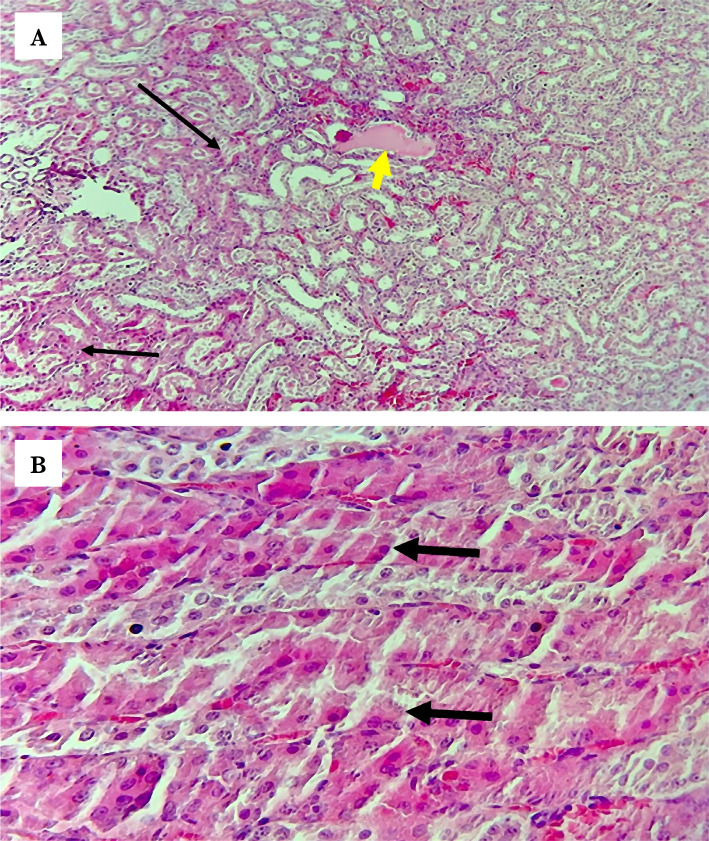
Representative photomicrographs of the LPS-RS group show (A) damage area (thin black arrows) with eosinophilic cast (yellow arrow), (B) severe damage with prominent cellular swelling and cytoplasmic eosinophilic (thick black arrows) H&E Magnification x100.

In the LPS-RS group, the abnormalities in the renal tissues were comparable to those in the non-treated control and vehicle groups. Severe ischemic damage in the renal tissues was found in the LPS-RS group, along with renal tubular cellular swelling, eosinophilic cast, and cytoplasmic eosinophilic (Figure 5 AB). The tissue injury score of the LPS-RS group was significantly elevated compared with the sham group and comparable to the scores of the control and vehicle groups, as shown in Table 1 and Figure 1. This means that LPS-RS does not have a renoprotective effect *versus* the renal IRI.

### ULPS-RS groups

**Figure 6:**
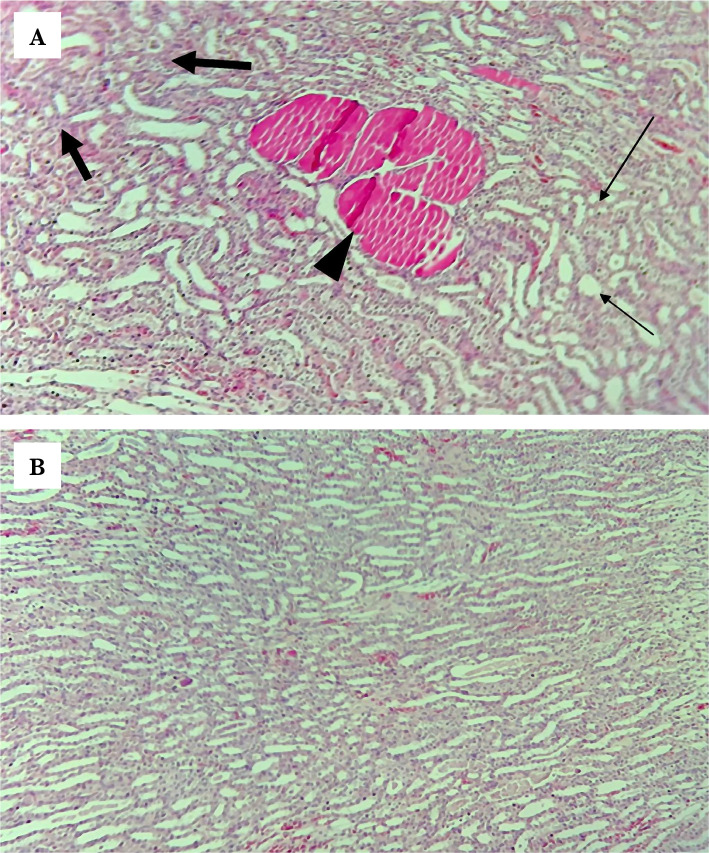
Representative photomicrographs of (A) the ULPS-RS group shows normal area (thin arrows) and damaged area (thick arrows) with eosinophilic cast (arrowhead), (B) the ULPS-RSH group exhibits renal tubules with normal histology H&E Magnification x100.

At both doses, ULPS-RS significantly improved the renal tissue injury scores compared with the control group (Table 1), suggesting renoprotective potential at these doses. Regarding the histopathological findings, mild changes were seen in the ULPS-RS group (0.1 mg/kg) compared with the control group (Figure 6 A), and the histological findings were even comparable to the sham group in the ULPS-RSH (0.2 mg/kg) (Figure 6 B).

### Effects of renal IRI, LPS-RS, and ULPS-RS on TLR4 expression

**Figure 7:**
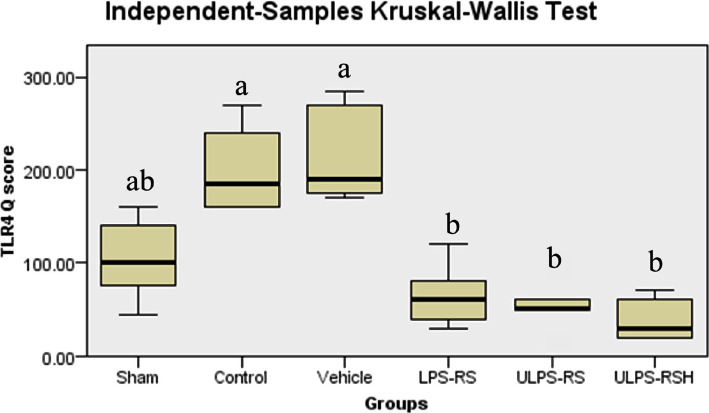
Boxplot showing the distribution of TLR4 Q scores across each group. Boxes with different letters are significantly different (P<0.05); boxes with letters (ab) do not significantly differ from boxes with letters (a) or (b).

The differences in TLR4 expression were evaluated using the Kruskal-Wallis H test. The test showed a significant difference in TLR4 expression between the different groups (test statistics=36.388, P<0.05), and the average ranks for the groups are shown in Table 1. The pairwise differences in Q scores for TLR4 IHC-P staining are summarized in Figure 7. The IHC-P staining results for TLR4 demonstrated highly upregulated TLR4 (though failed to be significant) in both the control and the vehicle groups compared to the sham group, as in Figures 7 and 8 (A–C). On the other hand, the LPS-RS, ULPS-RS, and ULPS-RSH groups showed significantly reduced TLR4 expression than the control group, as shown in Figures 7 and 8 (D–F). The variations in Q score for TLR4 IHC-P staining from the six experimental groups are shown in Table 1 and Figure 7.

**Figure 8:**
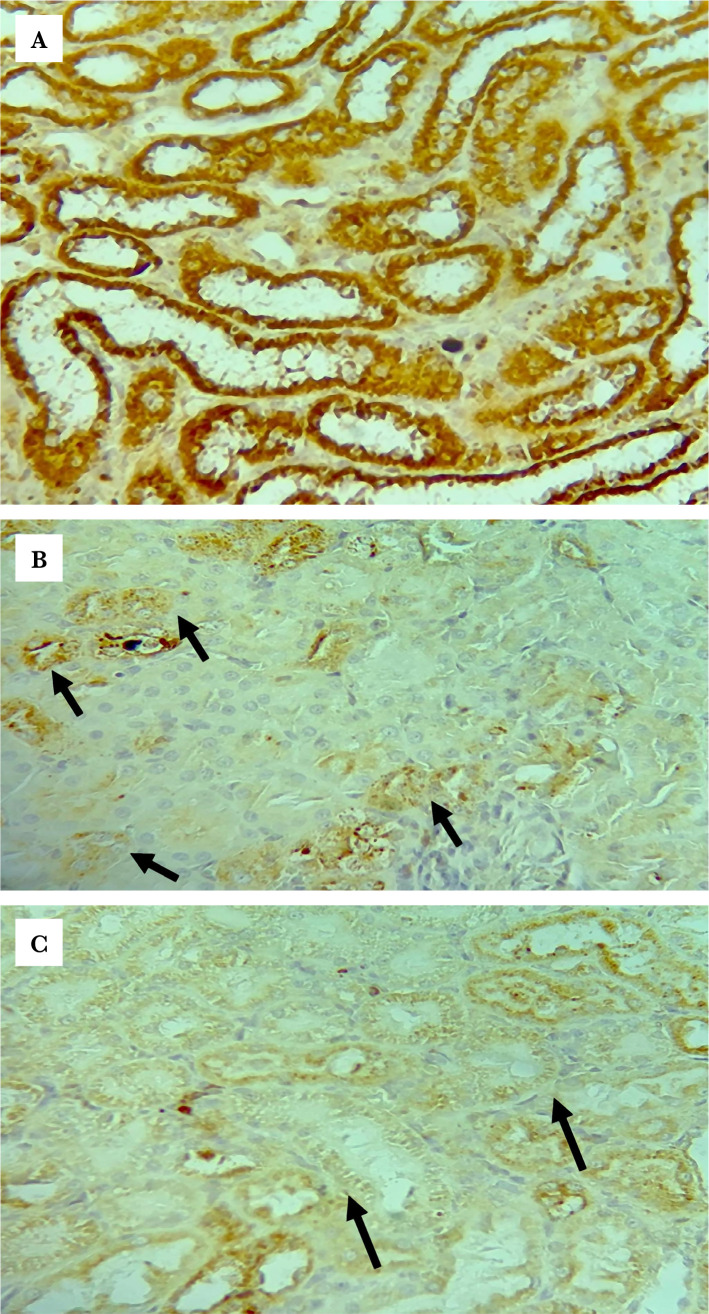
Representative photomicrographs of immunohistochemistry staining results for TLR4 in the (A) sham group shows focal weak brown staining (Black arrows); (B) control group shows strong brown staining (black arrows) in most of the renal tubules; (C) vehicle group shows diffuse strong brown staining; (D) LPS-RS group shows focal <15% of examined tissue weak brown staining (black arrows); (E) ULPS-RS group shows negative brown staining; (F) ULPS-RSH group shows focal weak brown staining (thin arrows for stained cells and thick arrow for negative cells), magnification x400.

**Figure 8:**
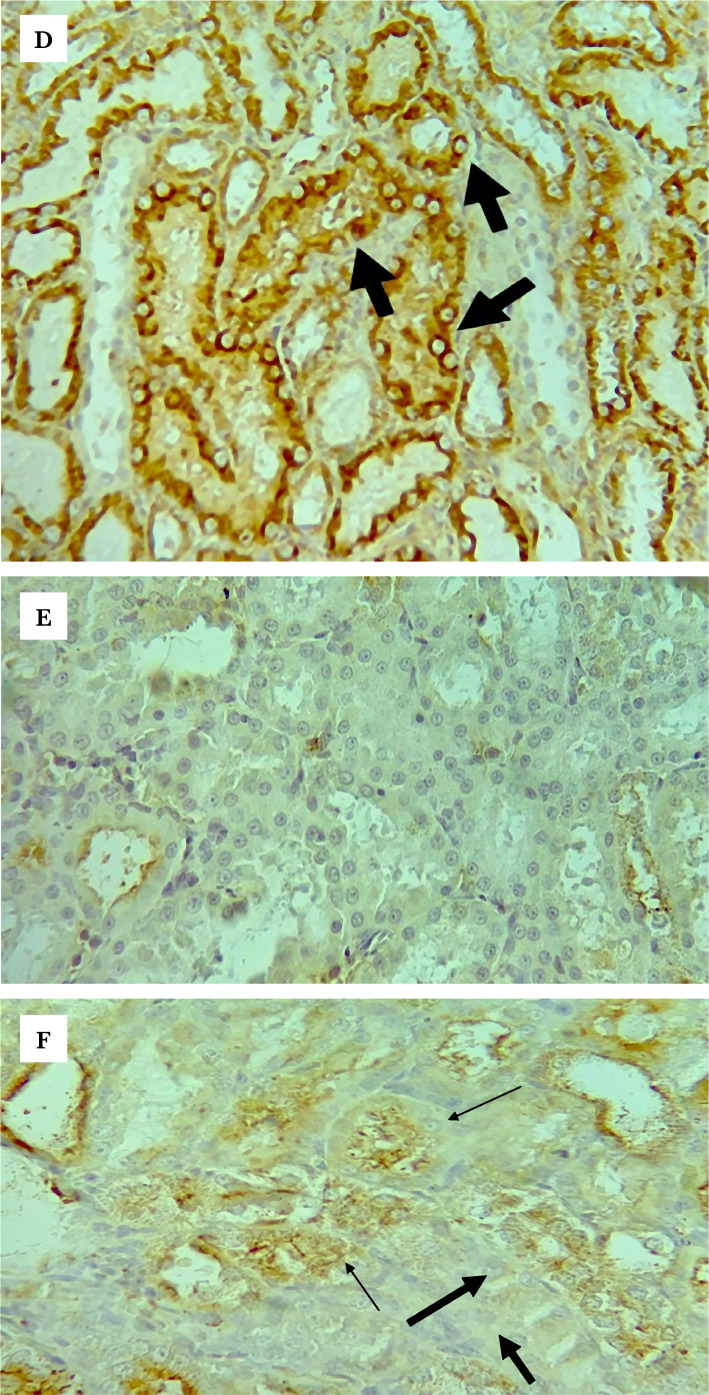
Continued.

### Effects of renal IRI, LPS-RS, and ULPS-RS on TLR2 expression

**Figure 9:**
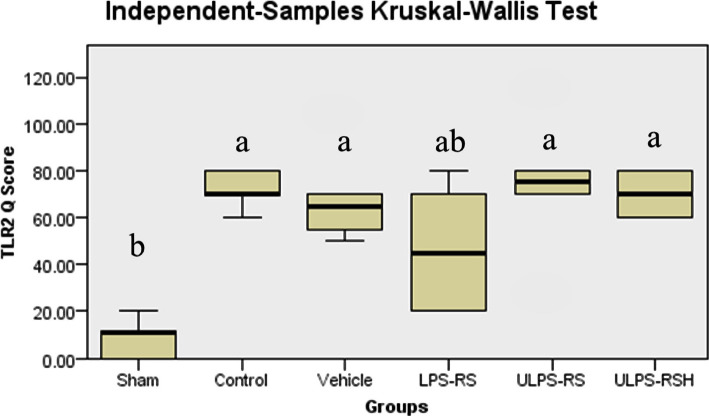
Boxplot showing the distribution of TLR2 Q scores across each group. Boxes with different letters are significantly different (P<0.05), and boxes with letters (ab) do not significantly differ from boxes with letters (a) or (b).

TLR2 expression differences between the six groups were also investigated. The Kruskal-Wallis test revealed significant differences in TLR2 Q scores between the different groups (test statistics=25.651, P<0.05), and the mean ranks for all groups are shown in Table1. The differences in TLR2 Q scores among the individual groups are summarized in Figure 9 and Table 1. TLR2 staining was significantly upregulated in the control, vehicle, ULPS-RS, and ULPS-RSH groups compared with the sham group. Additionally, the expression levels of TLR2 in the LPS-RS group were not significantly different from the other groups (Figure 9). The IHC-P staining results of the six experimental groups are shown in Figure 10 A–F.

**Figure 10:**
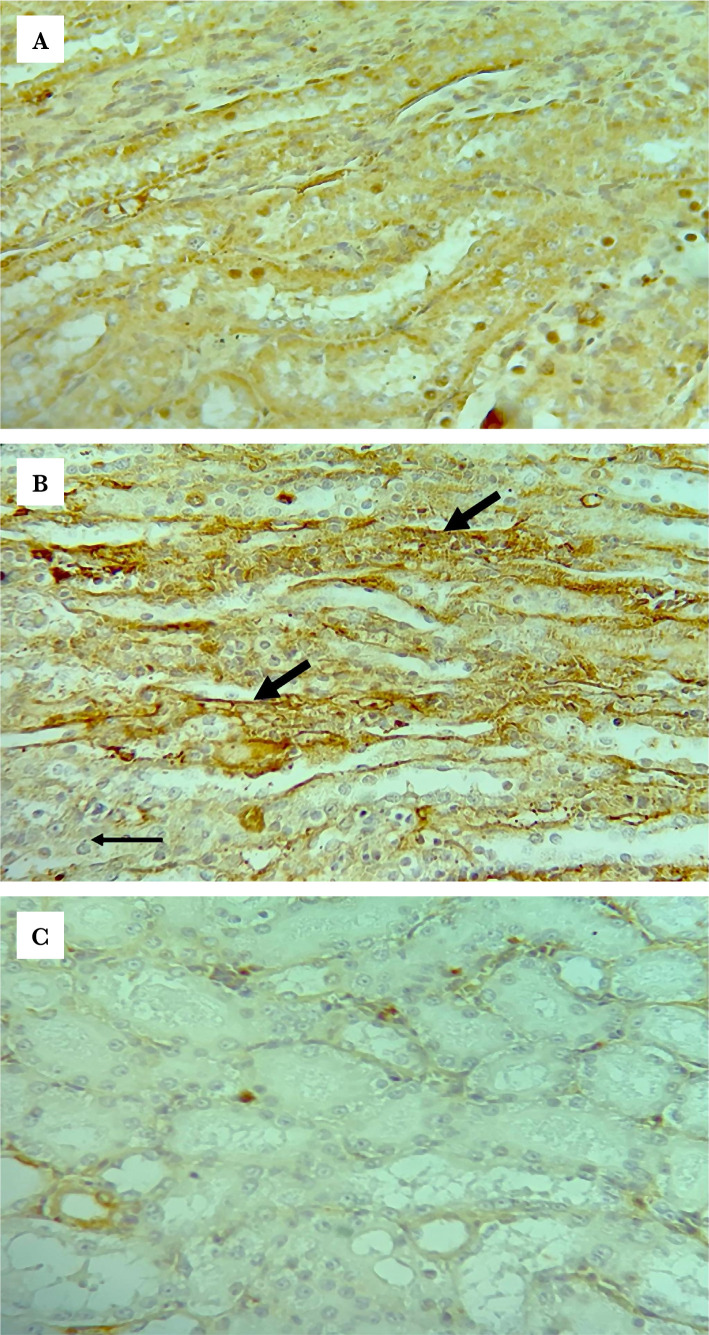
Representative photomicrographs of TLR2 immunohistochemistry staining results of (A) the sham group shows negative staining, (B) the control group shows moderate intensity brown staining, (C) the vehicle group shows positive brown staining, (D) LPS-RS group shows focal moderate intensity brown staining (thick arrows for stained cells and thin arrow for negative cells), (E) ULPS-RS group focal strong intensity brown staining (black arrows), (F) ULPS-RSH group focal weak intensity brown staining (black arrow), magnification x400.

**Figure 10:**
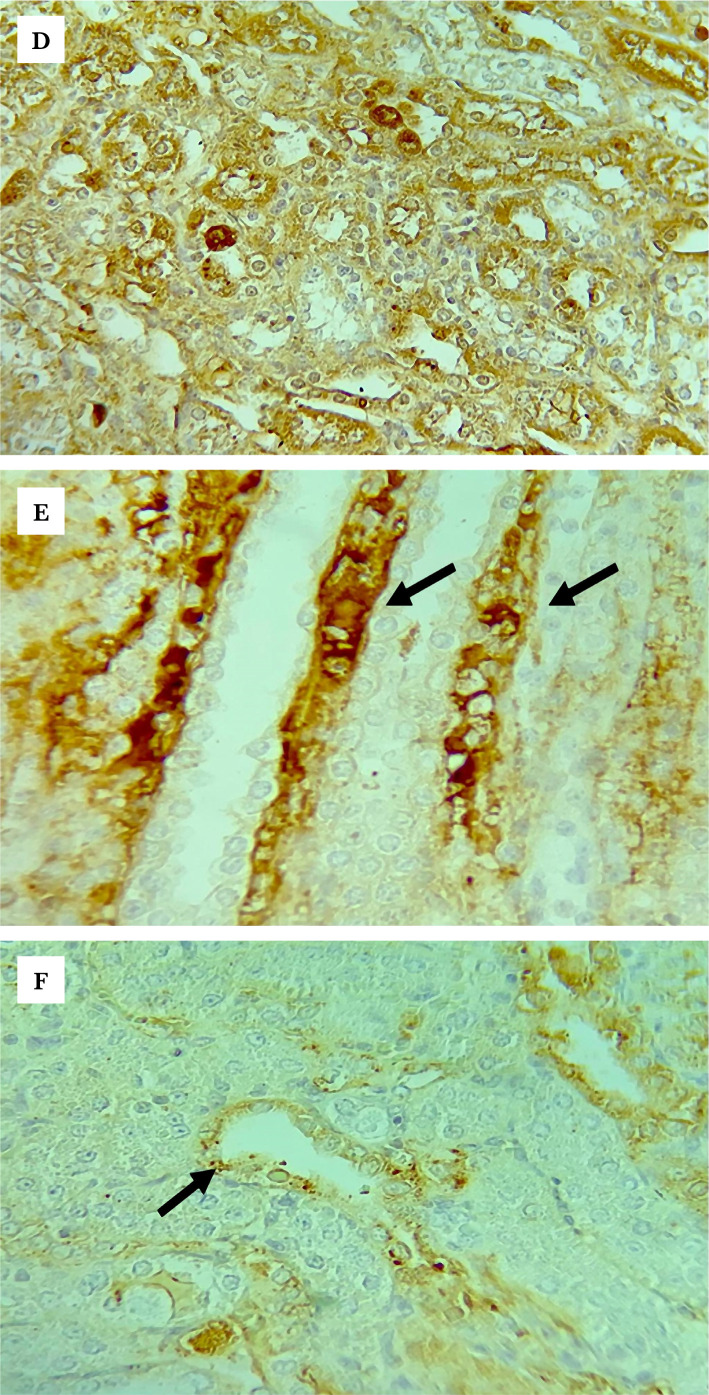
Continued.

## DISCUSSION

In the present study, we showed for the first time the effects of two compounds of bacterial origin on renal IRI in an animal model. Briefly, ULPS-RS at both doses was highly effective in reversing the renal tubular damages induced by IRI, as reflected by the histopathological findings of this study, and this effect was partially mediated by reducing TLR4 expression. Another highly interesting finding is that LPS-RS offered no protection against renal IRI at the tested dose even though it exhibits a similar effect on TLRs expression as ULPS-RS.

### Animal model

The animal model investigated in the current study has been thoroughly studied in several studies. We chose this ischemic time because it was shown that this duration (30 minutes) of ischemia was associated with significant AKI [18–31]. The reperfusion time was chosen because it was associated with severe renal injury, including defects in the renal excretory functions [32]. Male rats were chosen because of the testosterone-related higher susceptibility to kidney injury [33].

### Effect of renal IRI, LPS-RS, and ULPS-RS on renal parenchyma

In the current study, IRI of the kidneys resulted in severe damage to the renal tissues, as reflected by the histopathological scores of the control and vehicle groups. The tissue damages induced by the renal IRI include tubular epithelial swelling, necrotic tubules, brush border loss, cast formation, vacuolar degeneration, and desquamation. These results agree with previous studies [26, 34]. Alternatively, pretreatment with LPS-RS did not offer protection against renal IRI, while ULPS-RS offered significant protection *versus* IRI at both doses. Both compounds were demonstrated to block TLR4 in various tissues such as the lungs, the spinal cord, and dorsal root ganglia, and LPS-RS was also proposed to antagonize TLR2 in the latter tissues [22, 35]. Moreover, TLR4 inhibitors have been shown to protect from the damages induced by renal IRI, which agrees with the results of ULPS-RS but contradicts those of LPR-RS [16, 36], which indicates that other mechanisms could be responsible for the persistent renal damage seen after treatment with LPS-RS.

### Effect of renal IRI, LPS-RS, and ULPS-RS on toll-like receptor 4 expression

TLR4 protein expression was almost doubled within 2 hours after reperfusion in the control group compared with the sham group. Constitutive expression of TLR4 has been previously reported, and it explains the detection of TLR4 in the sham group [37]. Additionally, TLR4 expression was previously shown to significantly increase in response to renal IRI up to 9 days after the injury. Moreover, studies also found that TLR4 was crucially involved in mediating the renal IRI [38, 39]. On the other hand, LPS-RS and ULPS-RS significantly reduced the expression of TLR4 after IRI since both compounds were shown to block TLR4 [35, 40]. This could be how ULPS-RS exerts a renoprotective effect since previous studies demonstrated that TLR4 antagonists were effective as nephroprotective agents against IRI [16, 36]. Surprisingly, LPS-RS was associated with similar renal tissue injuries compared with the control groups, suggesting other mechanisms behind this detrimental effect.

### Effect of Renal IRI, LPS-RS, and ULPS-RS on toll-like receptor 2 expression

Significant upregulation ofTLR2 in response to IRI was found in the control, vehicle, and ULPS-RS groups compared with the sham group. TLR2 was reported to be constitutively expressed in different parts of the kidneys, and it was reported that TLR2 expression was upregulated in response to renal IRI [39, 41]. In the ULPS-RS groups, comparable expression patterns for TLR2 were seen compared with the control group, which means that the nephroprotective effect of ULPS-RS was solely through TLR4 and not through TLR2. This was expected since the ULPS-RS was reported in several studies to be a pure TLR4 antagonist [40–42]. However, LPS-RS pretreatment did not significantly reduce the expression of TLR2 in the current study compared with the control. This may be explained by the dose used in this study, as TLR2 was not significantly different from the sham group.

On the other hand, Jurga et al. [42] found that LPS-RS could block TLR2 in the spinal cord and dorsal root ganglia and thus attenuate pain in the rat neuropathic pain model [35], although they did not clearly mention the effect of treatment on TLRs expression. The proposed TLR2 blocking effect of LPS-RS failed to be significant in the present study which may be due to different dosing regimens used in our study since Jurga et al. used daily intrathecal dosing for seven days or may be due to the difference in the investigated organs. Both TLR4 and TLR2 were reported to mediate renal IRI, so antagonizing these receptors would be anticipated as more effective than purely antagonizing TLR4. However, LPS-RS failed to offer a renoprotective effect after IRI compared to the ULPS-RS, which was a highly effective renoprotection in the current study. This finding contradicts previous work in which a nephroprotective effect was shown after administering FTY720, associated with a reduction in TLR2 and TLR4 expression [17]. Accordingly, more work is required to explain why LPS-RS did not offer protection against IRI even though it blocks TLR4. Collectively, our results showed for the first time the nephroprotective effect conferred by a compound of bacterial origin and opened the door for more studies to further explain the interesting fact that is responsible for the lack of the protective effect observed after treatment with LPS-RS.

## CONCLUSION

Our results demonstrated that ULPS-RS was renoprotective while LPS-RS had no protective effect against the tissue damages induced by renal IRI.
